# Informant single screening questions for delirium and dementia in acute care – a cross-sectional test accuracy pilot study

**DOI:** 10.1186/s12877-015-0016-1

**Published:** 2015-02-25

**Authors:** Kirsty Hendry, Terence J Quinn, Jonathan J Evans, David J Stott

**Affiliations:** Institute of Cardiovascular and Medical Sciences, University of Glasgow, Glasgow, UK; Institute of Health and Wellbeing, University of Glasgow, Glasgow, UK; Academic Section of Geriatric Medicine, Institute of Cardiovascular and Medical Sciences, Room 2:03, 2nd floor New Lister Building Glasgow Royal Infirmary, Glasgow, G31 2ER UK; Academic Section of Geriatric Medicine, Institute of Cardiovascular and Medical Sciences, Room 2.44, 2nd floor New Lister Building Glasgow Royal Infirmary, Glasgow, G31 2ER UK; Mental Health and Wellbeing, University of Glasgow, The Academic Centre, Gartnavel Royal Hospital, R212 Level 2, 1055 Great Western Road, Glasgow, G12 0XH UK; Academic Section of Geriatric Medicine, Institute of Cardiovascular and Medical Sciences, Room 9, 2nd floor New Lister building Glasgow Royal Infirmary, Glasgow, G31 2ER UK

**Keywords:** Dementia, Delirium, Cognitive screening, Geriatrics, Psychology

## Abstract

**Background:**

Cognitive impairment often goes undetected in older people in hospital. Efficient screening tools are required to improve detection.

To determine diagnostic properties of two separate informant-based single screening questions for cognitive impairment (dementia and delirium) in hospitalised older people.

**Methods:**

Patients over 65 years non-electively admitted to medical or geriatric wards within a teaching hospital. Our index tests were single screening questions (SSQ), one for dementia (“How has your relative/friend’s memory changed over the past 5 years (up to just before their current illness)?”) and one for delirium (“How has your relative/friend’s memory changed with his/her current illness?”), which were assessed with informant response given on a five point Likert scale.

Any deterioration on our index tests of SSQ-dementia and SSQ-delirium was accepted as a positive screen for cognitive impairment. Scores were compared to the Informant Questionnaire for Cognitive Decline in the Elderly (IQCODE) *>*3.38 accepted as dementia, and Confusion Assessment Method (CAM) diagnosis of delirium. We also collected direct cognitive screening data using Mini Mental Status Examination (MMSE).

**Results:**

Informant responses were obtained in 70/161 (43.5%) patients, median age 80.8 (range:67–97) years; mean MMSE score 18.5 (SD: 8.1). The SSQ-dementia when compared to the IQCODE had a sensitivity of 83.3% and specificity of 93.1%. The SSQ-delirium when compared to CAM diagnosis had sensitivity of 76.9% and a specificity of 56.1%.

**Conclusions:**

These findings show promise for use of an informant single screening question tool as the first step in detection of dementia in older people in acute hospital care, although this approach appears to be less accurate in screening for delirium.

## Background

Cognitive impairment is a term covering a range of disorders representing a clinical deficit in cognitive ability with a significant deterioration from the person’s previous level of function [1]. It can present acutely with rapid onset and a fluctuating short-term course such as delirium, or as a chronic illness with a gradual, progressive course, for example mild cognitive impairment (MCI) and dementia.

Cognitive impairment is a strong predictor of negative outcomes such as increased length of hospitalisation, increased 6 month mortality and increased risk of readmission in older hospitalised individuals [2,3]. Cognitive impairment is commonly seen in the acute care setting, with between 40% and 70% elderly patients in acute care in UK hospitals having dementia with with less than half of these patients having a known previous diagnosis [4,5].

In-depth psychiatric assessment is not usually available as a first means of detecting cognitive impairment due to time and opportunity constraints. Instead, cognitive screening tests that are quick and easy to administer are used to identify individuals at high risk of cognitive impairment, suitable for further assessment [6]. Single question screening tests have been described for dementia [7] and delirium [8]. There is debate surrounding which screening test is the best to use due to the wide range available and lack of validation in hospital settings [9].

We aimed to pilot the performance of two single screening questions (SSQ), one for delirium and one for dementia, in hospitalised, elderly individuals as part of a secondary analysis of a previously collected data set. The SSQs were compared to a validated informant assessment of dementia; the 16 item Informant Questionnaire on Cognitive Decline in the Elderly (IQCODE) [10] and delirium diagnosis based on the Confusion Assessment Method (CAM) [11]. We hypothesised that single informant-based screening questions would be sensitive and specific in detecting dementia and delirium in hospitalised older people.

## Methods

### Participants

We carried out a prospective, observational pilot study of patients aged ≥65 years admitted to the acute medicine unit or geriatric assessment unit of an urban teaching hospital. Participants admitted to acute medicine between October-December 2004 were randomly selected, up to a maximum of 7 subjects per 24 hours (using tables of random numbers, and linking this to alphabetical order of patient name). Participants from the geriatric assessment unit were consecutive admissions (February-March 2005). A total of 161 patients were recruited; 80 from the acute unit and 81 from the geriatric unit. The acute medical unit admits patients of all ages, presenting with medical conditions requiring emergency hospital admission; the geriatric assessment unit admits older adults (age > 65) and preferentially selects those with complex co-morbidity; frailty; physical or cognitive decline.

Patient exclusions were Glasgow Coma Scale verbal component rated as none or sounds only, moderate-severe dysphasia (grossly impaired comprehension, unintelligible speech, or major difficulties in expression), non-English speaking, learning disability, major deafness or blind, or readmission of patient previously included in the study.

The patient’s capacity to provide consent was decided by a qualified independent doctor. Those judged as not to have the capacity to provide consent had written informed consent provided by their next of kin. The next of kin were provided with an information sheet to explain study objectives as well as the nature of patient participation.

Direct patient screening was performed within 36 hours of patient admission. Assessment included the MMSE as a screen for cognitive impairment and also the Confusion Assessment Method (CAM) as a measure for detecting delirium. The assessments were performed by a single trained observer, a senior medical student, who received formal one-to-one training in bedside cognitive assessment from an experienced consultant geriatrician.

Information was also obtained from patient medical records following cognitive assessment. This included demographic details such as age, sex and date of birth, current living arrangements and next of kin information. We described functional ability using an Instrumental Activities of Daily Living scale [12].

Participants’ next of kin were provided with a study pack which contained an introductory letter, an information sheet, two consent forms, the Informant Questionnaire on Cognitive Decline in the Elderly (IQCODE), the two SSQ’s for dementia and delirium and an envelope to return the completed consent form, IQCODE and SSQ’s. Study packs were either handed to the next of kin in person, with verbal instructions also being given by the researcher or, if this was not possible, posted to the home address. In cases where the next of kin was not available to complete the study, any relative or carer who had known the patient for a minimum of 5 years could complete the study.

The study was approved by the Scotland A Multi-centre Research Ethics Committee.

### Reference standards

The CAM [11] is a commonly used measure of delirium in hospitalised patients. The CAM consists of 9 operationalised criteria based on the DSM-IIIR. Observations made during direct cognitive testing as well as information obtained from nurse interview regarding fluctuating course and sleep-wake cycle are used to evaluate four components of patient cognition; acute onset and 1) fluctuating course, 2) inattention, 3) disorganised thinking and 4) altered level of consciousness. For delirium to be diagnosed by the CAM, criteria 1) and 2) must be present as well as either 3) or 4).

The 16 item IQCODE [10] is an informant-based questionnaire which asks relatives to consider changes in the patient’s abilities at certain activities within the last 10 years. Such items include “remembering where things are usually kept” and “learning new things”. Relative responses are given on a 5-point Likert scale ranging from “Much improved” to “Much worse. Average rating across all items is then calculated with a cut off of >3.38 accepted as indicative of possible dementia in this study.

The MMSE [13] is a multiple component screening tool which aims to measure 6 cognitive domains; orientation, registration, attention & calculation, recall, language and copying ability. The test is administered directly to the patient, usually by a member of the medical team. The test is scored out of a total of 30 with a score of <24 generally accepted as indication of possible cognitive impairment.

### Index tests

Two SSQs were developed as screens for dementia and delirium. Relative responses were measured on a 5-point Likert scale. The 5 response options to each question were;

- Much Improved/A Bit Improved/Hasn’t Changed Much/A Bit Worse/Much Worse
- The SSQ-delirium was;
- “How has your relative/friend’s memory changed with his/her current illness?”
- The SSQ-dementia was;
- “How has your relative/friend’s memory changed over the past 5 years (up to just before their current illness)?”

### Statistical methods

Data were analysed using SPSS version 19. Clinical and demographic information was examined using descriptive statistics. We compared subjects with and without an informant response.

For analysis of SSQ data, we used three categories: “much worse”; “bit worse” and “no decline” (which was a combination of “much better”, “bit better” and no change scores). To allow test accuracy analysis, SSQ responses were further dichotomised as suspected cognitive impairment (“bit worse” and “much worse” responses) and no cognitive impairment (“much better”, “bit better” and “no change” responses).

We used ROC analyses to compare index test of SSQ-delirium against the reference standard CAM and also the MMSE. We compared SSQ –dementia against the reference standard of IQCODE. We used usual diagnostic thresholds for IQCODE (mean score <3.38) and for MMSE (total score <24).

We described diagnostic metrics of sensitivity; specificity; positive and negative predictive value and corresponding 95% confidence intervals (95%CI).

We described differences in scores on ordinal reference standard tests (IQCODE and MMSE) for the three SSQ categories across both SSQ’s. Patient’s scores on the MMSE and IQCODE were analysed for statistical significance between the three SSQ outcomes using Kruskal-Wallis H Test analyses. Between group analyses were then carried out to determine where the statistical difference lay using Mann Whitney U post hoc analysis with Bonferroni correction.

## Results

The characteristics of the 161 participants recruited for this study are shown in Table 1, as well as separate analyses for patients with complete and incomplete single screening question data. SSQ’s were completed for 70 patients. There was found to be no significant difference between characteristics of respondents and non-respondents except in terms of age (p = 0.049).

**Table 1:** **Summary of characteristics of all patients with specification of respondents and non-respondents to the single screening questions**

	**All patients**	**Patients with informant**	**Patients with no informant**
	**(n = 161)**	**(n = 70)**	**(n = 91)**
**Mean age (years)**	79.6	80.9 *	78.6 *
	(range = 65–97)	(range = 67–97)	(range = 65–94)
**Male n (%)**	62 (38.5%)	27 (38.5%)	35 (38.5%)
**Living arrangements n (%):**			
**Alone**	80 (49.7)	32 (45.7)	48 (52.7)
**With spouse/other family**	66 (41.0)	31 (41.2)	35 (38.5)
**Sheltered accommodation**	8 (5)	4 (4)	4 (4)
**Nursing/residential care**	4 (2.5)	1 (1.4)	3 (3.3)
**Cognitive and functional assessments**			
**MMSE mean (SD)**	18.9 (7.7)	18.5 (8.1)	19.2 (7.5)
**MMSE <24 n (%)**	107 (66.4%)	45 (64.3%)	62 (68.1%)
**CAM positive n (%)**	27 (16.8%)	13 (18.6%)	14 (15.4%)
**IADL mean score (SD)**	8.7 (4.3)	9.1 (4.2)	8.4 (4.4)

*p = 0.049.

*Abbreviations: CAM* Confusion assessment method, *MMSE* Mini-mental state examination, *IADL* Instrumental activities of daily living.

Of the 70 patients who had data for both the dementia and delirium SSQ, 26 (37.1%) had a positive screen on both single questions, 25 (35.7%) had a negative screen on both single questions, 9 (12.9%) had a positive screen only for delirium and 10 (14.3%) had a positive screen only for dementia.

Kruskal-Wallis H Test analysis revealed a statistically significant difference in MMSE scores between the different SSQ-delirium outcomes (H(2) = 21.4 , p < 0.001) (see Table 2).

**Table 2:** **IQCODE and MMSE scores (means and S.D.) across SSQ acute and chronic responses**

**SSQ response**		**No change or better**	**Bit worse**	**Much worse**
**IQCODE**	**Acute SSQ**	3.4	3.9**	4.6**
		(SD = 0.6, N = 35)	(SD = 0.6, N = 25)	(SD = 0.5, N = 10)
	**Chronic SSQ**	3.2	3.9**	4.7**
		(SD = 0.4 N = 34)	(SD = 0.6 N = 27)	(SD = 0.4, N = 10)
**MMSE**	**Acute SSQ**	22.9	15.0**	12.0**
		(SD = 5.6, N = 35)	(SD = 8.6, N = 25)	(SD = 5.8, N = 10)
	**Chronic SSQ**	22.0	17.1*	10.1**
		(SD = 6.3, N = 34)	(SD = 7.9, N = 27)	(SD = 6.6, N = 10)

*Notes: Mann Whitney U post hoc analysis with Bonferroni correction.*

* = p < 0.01, ** = p < 0.001, compared to ‘nochange or better’.

*Abbreviations: MMSE* Mini-mental state examination, *IQCODE* Informant questionnaire on cognitive decline in the elderly, *IADL* Instrumental activities of daily living.

A statistically significant difference in MMSE scores was also found between the different SSQ-dementia outcomes (H(2) = 16.8, p < 0.001), with a median of 3.06 for patients identified as “no change or better”, 3.94 for patients identified as “a bit worse” and 4.88 for patients identified as “much worse”.

Kruskal-Wallis H Test analysis also revealed a statistically significant difference in IQCODE scores between the different SSQ-delirium outcomes (H(2) = 27.3, p < 0.001), with a median of 3.07 for those identified as “no change or better”, 3.75 for those identified as “a bit worse” and 4.85 for patients identified as “much worse”.

A statistically significant difference in IQCODE scores was also found between the different SSQ-dementia (H(2) = 41.2, p < 0.001), with a median of 3.06 for those identified as “no change or better”, 3.94 for patients identified as “a bit worse” and 4.88 for patients identified as “much worse”.

ROC analyses were carried out to calculate the sensitivity and specificity values of the SSQ-dementia and the SSQ -delirium, when comparing screening accuracy to a directly comparable routinely used screening instrument (IQCODE with a cut score of <3.38 and CAM positive diagnosis, respectively). Analysis revealed the SSQ-dementia (AUC = 0.882) had good diagnostic accuracy and SSQ-delirium (AUC = 0.665) had fair diagnostic accuracy. Sensitivity and specificity data are reported in Table 3.

**Table 3:** **Analysis of diagnostic accuracy**

	**Single question for acute confusion**	**Single question for chronic deterioration in memory**
	**CAM + ve**	**IQCODE <3.38**
**Area Under Curve (95% CI)**	0.67 (50.8-82.3)	0.88 (79.6-96.8)
Sensitivity of Single Question	10/13	35/42
(%, 95% CI)	(76.9)	(83.3)
Specificity of Single Question	32/57	27/29
(%)	(56.1)	(93.1)
Positive Predictive Value of Single Question	10/32	35/37
(%)	(28.6)	(94.6)
Negative Predictive Value of Single Question	32/35	27/24
(%)	(91.4)	(79.4)

*Abbreviations: CAM* Confusion assessment method, *MMSE* Mini-mental state examination, *IQCODE* Informant questionnaire on cognitive decline in the elderly.

## Discussion

The most prominent finding from this pilot study was the high sensitivity and specificity of the single screening question for dementia. This one-item screen performed at a similar level to the routinely used 16-item IQCODE. However, while the single screening question for delirium showed a similar sensitivity, it had low specificity. Almost half of individuals with normal cognitive functioning, as classified by CAM diagnosis, were identified by the single screening question having suspected delirium.

While the SSQ-delirium appeared not to perform well as a first step in delirium detection, this may be better explained by methodological issues with difficulties in screening for delirium, in general. A defining feature of delirium is fluctuation in presence of symptoms, and as such it is very difficult to have exact concurrence between a screening tool for delirium and the reference standard; in this study the SSQ-delirium and CAM, respectively. Thus, it is possible that delirium can be present at one testing point but not the other. Interpretation of the performance of the SSQ-delirium in this study is limited as data on the time lag between the SSQ-delirium and CAM were not collected. It would also be of interest for future studies to test the SSQ-delirium blinded from CAM diagnosis.

Undiagnosed dementia may be able to account for the high number of false positive results identified by the single screening question for delirium. The majority of patients identified as positive by the single screening question for delirium also had a positive results on the single screening question for dementia. It is possible that an informant based question is not suitable to accurately differentiate those at high risk of having delirium from those at high risk of having dementia.

Direct cognitive testing of patients is the most commonly used screening method for cognitive impairment [14]. However, informant testing shows promise in improving detection of at-risk individuals. It has been demonstrated that an informant questionnaire shows the same performance as direct cognitive testing, despite the fact that these different screening tools measure different patient attribute [15]. A major advantage of informant based assessments is that they do not suffer the same problems as cognitive testing of being independent of patient’s education level and less susceptible to ceiling effects [16]. Single question screening for cognitive impairment is a hot topic at the moment and there is a paid incentive being rolled out across hospitals in England in an attempt to improve diagnosis of patients with cognitive impairment known as the Commission for Quality and Innovation (CQUIN) framework. Of particular interest to this study is the first stage, ‘Find’ whereby the patient or informant is asked the question, ‘Has the patient been more forgetful in the last 12 months to the extent that it has significantly affected their daily life?’ This suggests that single question methods of screening for cognitive impairment are beginning to be used on a large scale despite not being fully validated.

The advantage of using an informant-based screening tool for delirium is less clear than for dementia. Due to the fluctuating nature of delirium, it can easily be missed and the need for a relative to be present to provide information introduces further timing challenges as access to informants is only possible at discrete selected times. However, evidence suggests that delirium is most prevalent in older patients when they are at their most sick, usually soon after admission or in those patients who have prolonged hospitalisation. Thus, those may be particularly important times to use the SSQ-delirium. It is clear from the literature that some form of delirium screening is needed rather than subjective clinical judgement which has been shown to perform poorly at detecting prevalent delirium [17]. This supports the need for a more structured approach to be implemented as a brief first step to identify those with suspected delirium who would then be assessed using diagnostic tools such as the CAM.

Caution must be taken when interpreting these results as the reference standards were screening tests rather than a formal clinical diagnosis. lack of comprehensive clinical evaluation for dementia was a further major limitation . The recruitment and consent strategy could have lead to biases within this study; those at higher risk of cognitive impairment may have had more visits from family and hence more likely to return the informant questionnaire, especially in cases where the patient was unable to provide consent and hence a relative or carer had to provide consent. As this study obtained single question informant report in less than half of patients this raises issues of feasibility as there is strong potential for many individuals with suspected cognitive impairment not being assessed. From this data set, it is unclear whether such low response rates were due to patients not having a suitable relative or carer available to answer the single screening questions or whether it was due to a lack of appropriate measures taken by the researcher to insure return of informant responses.

Presenting informants with the IQCODE prior to answering the single screening questions may also have influenced the results, possibly enhancing how well the single screening questions appeared to perform.

However, within these limitations we believe our results still provide useful information on how simple responses from informants may perform. This pilot study was strengthened by broad inclusion criteria thus providing a sample relatively representative of older adult acute care admissions. We based our SSQ’s on the format of a validated informant questionnaire, the IQCODE.

These preliminary findings show promise for use of a single question screening tool as the first step in the detection of cognitive impairment and prompt more thorough investigation. As yet there is no one cognitive screening tool that has achieved widespread consensus, thus the comparisons made to the IQCODE and MMSE only provide evidence for the value of carrying out a more in-depth study. Future research should compare the single question screen to a gold standard clinical evaluation of dementia and delirium to determine more reliable diagnostic accuracy figures. The use of an informant-based single screening question may be particularly useful in combination with a direct cognitive testing method in helping to distinguish patients who fall within the middle, grey area of scores on cognitive test based screening tools. Furthermore, it is apparent that there is need for investigation to determine how to get higher uptake of relatives/carers to provide informant report.

## Conclusions

Routine screening for cognitive impairment is necessary in older hospitalised patients and effective screening is beneficial to clinical staff as well as the outcomes of the patients. This task is made more difficult by the lack of uniformity regarding the various screening tools available [18]. This pilot study has provided evidence that a single-item informant based screen may perform in a comparable way to much longer screens for dementia by effectively differentiating those with suspected cognitive impairment from normally functioning patients. Further validation is warranted. However, if a screening tool as straightforward as asking a single question, which has low time cost and requires little to no training to administer, can perform as well as more complex screens such as the IQCODE, then it would seem intuitive that this is a preferable option. However, results showed that a single question screening tool may not have the same potential when identifying individuals with suspected delirium.
